# Starch composition, glycemic indices, antioxidant properties and carbohydrate hydrolyzing enzymes activities of African star apple fruit parts

**DOI:** 10.1186/s12906-020-03053-9

**Published:** 2020-08-25

**Authors:** Olubunmi B. Ajayi, Folake L. Oyetayo, Seun F. Akomolafe

**Affiliations:** Department of Biochemistry, Ekiti State University, Ado Ekiti, Ekiti State P.M.B. 5363 Nigeria

**Keywords:** African star apple fruit, α-Amylase, Diabetes mellitus, α-Glucosidase, Antioxidant, Acarbose

## Abstract

**Background:**

African star apple (*Chrysophyllum albidum*) is a traditonal fruit, which is predominant in tropical regions with the fruit parts consumed by the populace and used in folklore to manage diabetes. However, the likely activity mechanism is still undetermined. The current study examined and compared the inhibitory abilities of African star apple fruit parts on selected key enzymes related to diabetes mellitus in the pancreas tissue of rat.

**Methods:**

Inhibitory effect of aqueous extract (1:10 w/v) of African star apple fruit parts (pulp, cotyledon, seed coat and pulp coat) on the activities of α-amylase, α-glucosidase, as well as their starch composition, phenolic constituents, estimated glycemic index, and antioxidant properties were assessed.

**Results:**

The fruit parts showed low sugar, eGI, amylose, and amylopectin contents. The analysis also showed that the fruit parts inhibited α-glucosidase and α-amylase activities and exhibited antioxidant properties. Furthermore, the fruit parts contain high concentrations of beta-amyrin acetate, eleagine, epicatechin, epigallocatechin, skatole, stigmasterol and tetrahydro − 2- methylharman as revealed by HPLC-DAD.

**Conclusion:**

The fruit part low estimated glycemic indices, strong antioxidant properties, inhibition of α-amylase and α-glucosidase activities exhibited might be related to the bioactive compounds contained in the extract. This could also be a potential mechanism for the use in the prevention and management of type-2 diabetes. Nevertheless, the African star apple pulp coat displayed the highest property in comparison to other parts of the fruit.

## Background

The inability of β-pancreatic islet to produce sufficient insulin or un-utilization of insulin by the body is an underlining pathology of Diabetes mellitus. This civic medical challenge is linked to the rise in the rates of mortality and morbidity globally [1]. Nevertheless, administrating carbohydrate hydrolyzing enzymes (α-glucosidase and α-amylase) inhibitors (like acarbose) and/or reduction of carbohydrate consumption can decrease the absorption of glucose into the blood stream [2, 3]. This will help to reduce glucose assimilation into the blood stream [3]. Acarbose, which is a synthetic drug currently in use for the inhibition of carbohydrate hydrolyzing enzymes has been reported to unveil several adverse influences. Herbs have been used by man from time immemorial to prevent and/or treat a wide array of disorders. Plant derived substances and/or plant extracts are naturally preferred by most individuals because they have lesser side effects and are cheaper, safer and tends to have better good health promotion than most synthetic drugs [2, 4–6].

The consumable part of the plants regarded to as fruits are available commercially as food which could be consumed in the raw form or processed into wine, and fruit juices [7].. Processing fruits generates large amounts of waste products which, according to reports, are rich in antioxidant [8]. In the tropics, especially Nigeria, a huge part of the country and notable for various fruits production, fruits are relished throughout the year. Oboh et al. [9] reported that the common fruits expended in Nigeria are orange, pineapple, African star apple, watermelon, pawpaw, mango, banana, carrot, cashew, among others.

African star apple (*Chrysophyllum albidum* G. Don (Sapotaceae)) is a traditional fruit which is predominantly found in Africa. The fruit is a lowland rain forest tree species which belongs to the family of Sapotaceae. It grows up to 25 to 37 m in height, with its girth between 1.5 to 2 m [10]. The fleshy pulp is widely consumed and used to manage diabetes in folklore [11]. In folkloric medicine, the plant bark is valuable in treating malaria and yellow fever [11], while the leaf has been used for treating stomach ache and diarrhea as well as a palliative. The leaves, barks, and roots are widely used for treating wounds, sprains, and bruises in the southern part of Nigeria. The root and seed extracts are useful in arresting bleeding from fresh wounds and helps to prevent wound contamination thereby, hastening the healing process [12]. According to report, the seed cotyledon exhibits both hypolipidemic and anti-hyperglycemic effects [13]. The fruit pulp can be freshly consumed and processed into stewed fruit, syrup, jam, soft drinks, and jellies. According to Akubugwo and Ugbogu [14], the fruit pulp of African star apple is highly rich in iron and ascorbic acid compared to other edible fruits. Oboh et al. [15] reported that the different parts of the fruit are potentially rich sources of functional foods and nutraceuticals which have neuroprotective properties due to their polyphenolic constituents and cholinesterases and monoamine oxidase activities, as well as their antioxidant properties. Ibrahim et al. [16] documented variations of phytochemicals and essential nutrients in edible parts of *Chyrsophyllum albidum* fruit, and afterward presumed that because of the phytochemicals and basic supplements as dissolvable and starch, insoluble fibers, arabinose and mineral components found in the pulp coat of *Chrysophyllum albidum* in correlation with the seed coat, cotyledon and fruit pulp has an incredible potential in adding to the sound growth and as supplementation in food industries. Abiodun and Oladapo [17] additionally detailed that the pulp coat and seed pericarp of the fruit which are generally disposed of as waste items during juice processing have been appeared to contain higher nutritive constituents than pulp part.

Also, in our previous study, we reported that the dietary supplementation of African star apple fruit pulp powder attenuated hyperglycemia and oxidative stress by altering biochemical parameters in type 2 diabetic condition [2]. There are no reported investigations into the toxicity activity of different parts of *Chrysophyllum albidum* fruit, except the butanolic extract of the seed cotyledon which has been reported by Shobo et al. [18] to cause mild to moderate toxicity at higher portions, and and subsequently inferred that while exploiting its numerous economic and therapeutic properties, there is the need to protect against aimless utilizations and to apply caution in the use of the seed cotyledon of *Chrysophyllum albidum*.

**Fig. 1:**
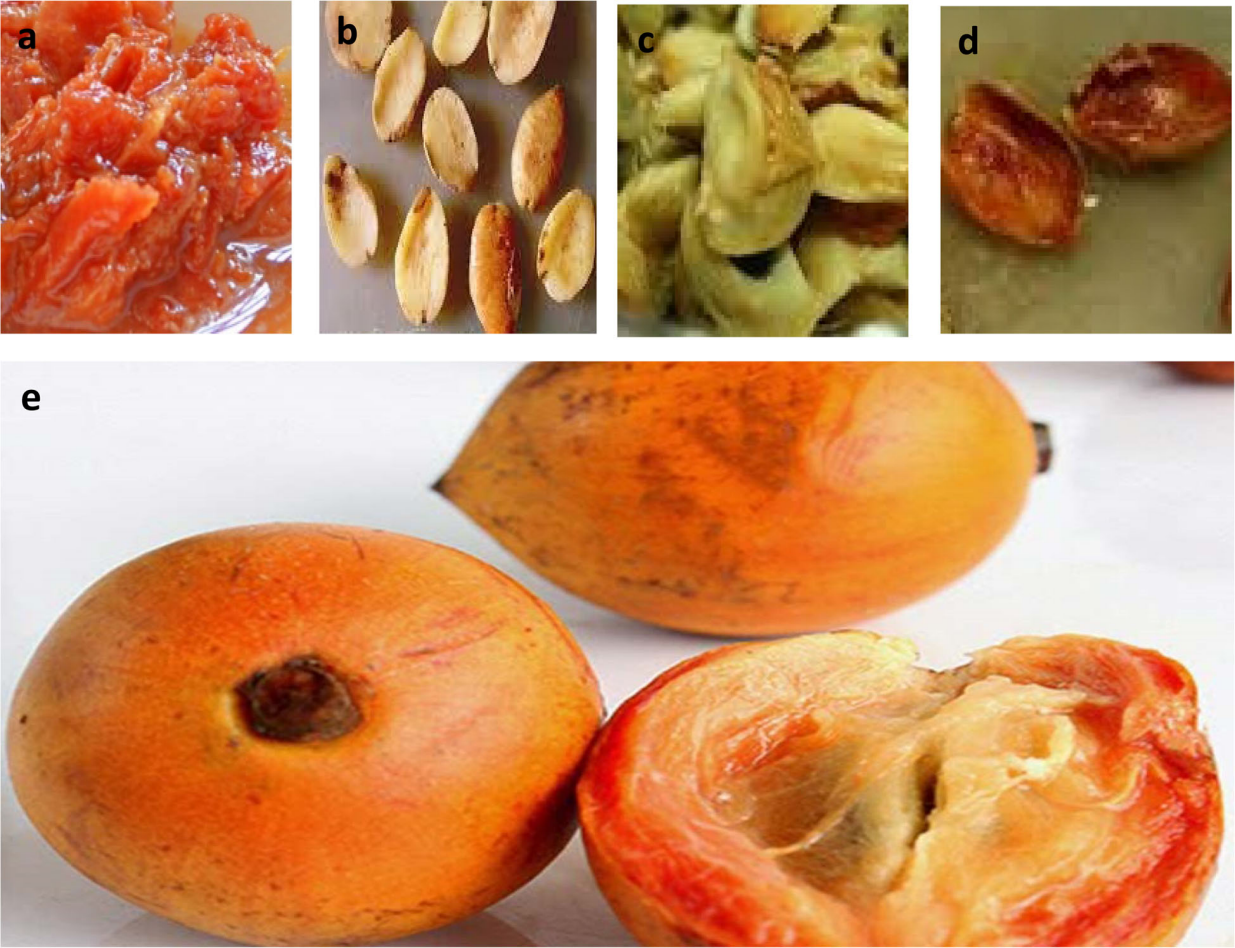
African star apple fruit parts (**a**) Pulp (**b**) cotyledon (**c**) Seed coat (**d**) Pulp coat (**e**) Fruit

Additionally, previous reports have revealed the phenolic constituent and antioxidant properties of African star fruit [15, 19, 20], but to the best of our knowledge, starch composition, estimated glycemic index (eGI), possible in vitro anti-hyperglycemic function of different parts of African star apple, and the likely biochemical mechanism that confirms their aptness as functional foods for managing of type-2 diabetes are yet to be elucidated. Accordingly, the current study aims to compare African star apple fruit parts, the pulp, cotyledon, seed coat and pulp coat (Fig. 1) as a possible source of functional foods and nutraceuticals with anti-hyperglycemic function by investigating the effect of aqueous extract of African star apple fruit parts on α-amylase and α-glucosidase activities, as well as their starch composition, estimated glycemic index, phenolic constituents and antioxidant properties.

## Methods

### Sample collection and identification

The ripe African star apple fruits were purchased in March, 2018 from the Erekesan market in Ado Ekiti metropolis, Ekiti State, Nigeria. Authentication of the sample was carried out by Mr. Omotayo at the Department of Plant Science and Biotechnology, Ekiti State University, Ado-Ekiti where voucher specimen (voucher ID number UHAE 667) was deposited at the herbarium of the same Department.

### Aqueous extract preparation

In order to eliminate any contaminants, the ripe African star apple fruits were thoroughly washed with clean water. The fruit parts (pulp, cotyledon, seed coat and pulp coat) were then separated and distinctly blended with distilled water (1:20 w/v), centrifuged and filtered. The clear supernatant obtained after filtration was then freeze-dried and placed in the refrigeration for subsequent analysis [21]. The fruits parts yield were 152.1 g dry sample/1000 g fresh fruit pulp, 126.5 g dry sample/1000 g fresh fruit cotyledon, 67.5 g dry sample/1000 g fresh fruit seed coat and 101.9 g dry sample/1000 g fresh fruit pulp coat for pulp, cotyledon, seed coat and pulp coat respectively. The freeze-dried fruit extract was then refrigerated at -10 °C and reconstituted for additional analysis.

### Determination of total flavonoid, total phenol, vitamin C contents and ferric reducing antioxidant property

The study followed the procedure of Adefegha et al [22] in obtaining the total phenolic constituent and the total flavonoid composition of the extract. To calculate the total flavonoid composition, quercetin was used as standard. To obtain the total phenolic content, gallic acid was employed as the standard. Thus, the content was obtained as gallic acid equivalent (GAE). The procedure applied by Akomolafe and Ajayi [23] was used in obtaining the vitamin C composition of the extract. The composition was calculated in equivalence to standard ascorbic acid solution. Following the procedure of Akomolafe et al. [24], the samples’ reducing property (based on the extract’s capacity to reduce FeCl_3_ solution) was determined and calculated in equivalence to standard ascorbic acid solution.

### Determination of radicals (ABTS, DPPH, OH and NO) scavenging and Fe^2+^-chelating abilities

Following the methods of Ademiluyi et al. [3] and Adefegha et al [22], two models were used in determining the sample’s capacity to scavenge free radicals. These are the 1, 1-diphenyl-2 picrylhydrazyl (DPPH) [25] and 2,2′-azino-bis (3-ethylbenzothiazoline-6-sulphonic acid) (ABTS) [3]. The samples’ Fe^2+^ chelating ability was assessed using the procedure of Akomolafe et al. [26]. In evaluating the nitric oxide scavenging activity of the extracts, the method of Akomolafe et al. [27] was followed. To determine the extracts’ ability to scavenge hydroxyl radical, the procedure of Halliwell et al [28] was followed.

### Experimental animals

Two adult male albino Wistar rats that weighed 202–210 g each were bought from the Department of Veterinary Medicine breeding colony, University of Ibadan, Nigeria. The rats were acclimatized at 25 °C, on a 12 h light/12 h dark cycle for a fortnight ahead of the experiment (lights on from 19:00 to 7:00 h). In handling the rats, NIH Guide for the care and use of laboratory animals and the ethical Committee for Animal Experimentation of the University were strictly followed. The study was approved by Ekiti State University ethical committee with reference number AOO012BAJO01. All experimental procedures were performed according to the ARRIVE guidelines [29].

### Preparation of tissue homogenate and lipid peroxidation assay

The procedure of Akomolafe et al. [30] and Akomolafe and Ajayi [23] were slightly modified and followed to prepare the pancreas homogenate and determine of thiobarbituric acid reaction assay. Two rats were placed under low doses of ketamine (10 mg/kg) and xylazine (10 mg/kg) anaesthesia administered via intraperitoneal (i.p.) injections, and euthanized by cervical dislocation, the rats were rapidly dissected. This was followed by rapid removal of the tissue, pancreas and washing with cold saline. The supernatant obtained from pancreatic tissue after preparation was used for the thiobarbituric acid reaction assay.

### α-Glucosidase and α-amylase activities assays

To evaluate the impact of the extract on α-glucosidase and α-amylase activities, the methods utilized by Dada et al. [31] was adopted. Utilizing acarbose drug as a standard control, the extract’s enzyme inhibitory impacts were expressed as percentage inhibition. This was determined after Equation (1):

1 $$ \mathrm{A}=\left[{\mathrm{ABS}}_{\mathrm{ref}}-{\mathrm{ABS}\mathrm{s}}_{\mathrm{ample}}\right]/{\mathrm{ABS}}_{\mathrm{ref}}\times 100 $$

### Sugar and starch determination

Starch and sugar analyses were performed utilizing the method of Onitilo et al. [32]. The starch and total free sugar contents of the sample were determined from a glucose standard curve prepared alongside the sample.

### Determination of the amylose and amylopectin content

Amylose and amylopectin contents were determined utilizing the method of Juliano [33]. Amylose content was determined utilizing standard amylose. Amylopectin was determined after eq. (2) [33]:

2 $$ \mathrm{amylopectin}=\mathrm{starch}\ \mathrm{value}-\mathrm{amylose}\ \mathrm{value} $$

### Estimation of glycemic index

The procedure applied by Brouns et al. [34] was utilized in obtaining the estimated glycemic index of the extract.

### HPLC analysis of phenolic components

The method clarified by Kelley et al. [35] was utilized in evaluating the quantitative–qualitative examination of the phenolic components of the samples. To quantify it, peak regions were associated with concentrations as per the calibration curves. Results are accounted for as means ± standard deviations of triplicate independent analyses.

### Data analysis

The experiments conducted in triplicate were pooled and presented as mean ± standard deviation. Comparison of Means was through a one-way ANOVA followed by Duncan’s multiple range test. The least significant difference tests were conducted and accepted at *p* ≤ 0.05.

## Results

### The sugar, amylose contents, starch, amylopectin contents, glycemic index and amylose/amylopectin ratio, of African star apple fruit parts

The free soluble starch contents (Table 1) of the fruit’s parts ranged from 3.13 g/100 g (pulp) to 4.42 g/100 g (cotyledon) while the free soluble sugar (Table 1) ranged from 21.48 g/100 g (cotyledon) to 28.63 g/100 g (pulp) (Table 1). The amylose content of the fruits samples ranged from 2.01 g/100 g (pulp coat) to 2.31 g/100 g (cotyledon) (Table 1) while amylopectin content of the pulp coat of the fruit is lowered in comparison to other parts. The amylose/amylopectin ratio of the fruit parts ranges from 0.37 to 0.44. The fruit parts had estimated glycemic indices ranging from 13.63 (pulp coat) to 29.10 (cotyledon) (Table 1).

**Table 1:** The starch, sugar, amylose and amylopectin contents, amylose/amylopectin ratio, and glycemic index of African star apple fruit parts

Sample	Pulp	Cotyledon	Seed coat	Pulp coat
Starch (g/100 g)	3.13 ± 0.05^*a*^	4.24 ± 0.27^*b*^	3.79 ± 0.03^*a*^	3.58 ± 0.13^*a*^
Sugar (g/100 g)	28.63 ± 0.00^*a*^	21.48 ± 0.04^*b*^	23.97 ± 0.02^*b*^	21.66 ± 0.03^*b*^
Amylose (g/100 g)	2.03 ± 0.03^*a*^	2.31 ± 0.02^*a*^	2.28 ± 0.03^*a*^	2.01 ± 0.02^*a*^
Amylopectin (g/100 g)	5.48 ± 0.02^*a*^	5.25 ± 0.02^*a*^	5.56 ± 0.00^*a*^	5.15 ± 0.05^*a*^
Estimated glycemic index (%)	15.79 ± 1.89^*a*^	29.10 ± 0.67^*b*^	20.21 ± 1.60^*c*^	13.63 ± 0.42^*a*^
Amylose/amylopectin ratio	0.37	0.44	0.41	0.39

Values represent mean ± standard deviation (*n* = 3). Values with the same superscript letter within a row are not significantly (*p* < 0.05) different

### In-vitro antioxidant property of African star apple fruit parts

Total phenolic content (Table 2) of fruit parts ranged from 7.56 mg GAE/g (cotyledon) to 23.01 mg GAE/g (pulp coat) while the total flavonoid content (Table 2) ranged from 2.27 mg QE/g (cotyledon) to 5.79 mg QE/g (pulp coat). The pulp coat had the highest vitamin C content (13.03 mg AAE/g) compared to other fruit parts. The ability of the fruit parts to scavenge ABTS radical revealed that the pulp had the least value (0.0370 mmol. TEAC/g) while the cotyledon had the highest value (0.0548 mmol. TEAC/g) (Table 2). The reducing power of the fruit parts can be ranked in the order, pulp coat (15.82 mg AAE/g) > pulp (10.23 mg AAE/g) > seed coat (9.75 mg AAE/g) > cotyledon (5.52 mg AAE/g) (Table 2). The result showed that the fruit parts scavenged the DPPH, OH* and NO* free radicals in a dose dependent manner (Table 3). Judging by the IC_50_ values, the DPPH scavenging ability of the fruit parts range from 1.32 mg/mL to 3.70 mg/mL with seed cotyledon (IC_50_ = 3.70 ± 0.57 mg/mL) having the least scavenging ability while pulp coat (IC_50_ = 1.32 ± 0.12 mg/mL) had the highest (Table 3). Hydroxyl (OH*) radical scavenging ability of the fruits parts showed that the pulp coat (IC_50_ = 3.69 mg/mL) had the highest OH* radical scavenging ability and pulp (IC_50_ = 6.12 mg/mL) the least (Table 3). The pulp coat (IC_50_ = 0.39 mg/mL) had the highest NO radical scavenging ability while the pulp exhibited the least ability (IC_50_ = 0.67 mg/mL). Also, pulp coat (IC_50_ = 0.14 mg/mL) had the highest iron chelating ability and fruit pulp (IC_50_ = 0.38 mg/mL) the least (Table 3).

**Table 2:** Phytochemical contents, ABTS scavenging ability and Ferric reducing antioxidant property of African star apple fruit parts

Samples	Pulp	Cotyledon	Seed coat	Pulp coat
Total phenolic content (mg GAE/g)	14.11 ± 2.74^*a*^	7.56 ± 2.74^*b*^	12.26 ± 2.84^*c*^	23.01 ± 2.65^*d*^
Total flavonoid content (mg QE/g)	5.31 ± 0.05^*a*^	2.27 ± 0.37^*b*^	3.69 ± 0.18^*a*^	5.79 ± 0.05^*a*^
Vitamin C content (mg/g)	7.26 ± 0.11^*a*^	5.48 ± 0.17^*a*^	5.54 ± 0.28^*a*^	13.03 ± 0.04^*b*^
ABTS scavenging ability (X 100) (mmol. TEAC/g)	3.70 ± 0.03^*a*^	4.00 ± 0.04^*a*^	3.97 ± 0.02^*a*^	3.87 ± 0.03^*a*^
Ferric reducing antioxidant property (mg AAE/g)	10.23 ± 0.02^*a*^	5.52 ± 0.58^*c*^	9.75 ± 0.00^*c*^	15.82 ± 1.14^*b*^

Values represent mean ± standard deviation (n = 3). Values with the same superscript letter within a row are not significantly (*p* < 0.05) different. *GAE* Gallic acid equivalent, *QE* Quercetin equivalent, *TEAC* Trolox equivalent antioxidant capacity, *AAE* Ascorbic acid equivalent

**Table 3:** Effective concentration causing 50% antioxidant ability (IC_50_ values) of 1,1-diphenyl-2 picrylhydrazyl (DPPH), hydroxyl (OH), nitric oxide (NO) radical scavenging abilities, iron chelating ability of fruits parts

Sample	DPPH*(mg/Ml)	OH*(mg/ml)	NO*(mg/ml)	Fe^2+^ chelating ability (mg/ml)
Pulp	3.03 ± 0.48^*b*^	6.12 ± 2.07^*a*^	0.67 ± 0.23^*a*^	0.38 ± 0.10^*a*^
Cotyledon	3.70 ± 0.57^*c*^	5.63 ± 1.77^*b*^	0.40 ± ±0.26^*b*^	0.26 ± 0.15^*b*^
Seed coat	3.60 ± 0.55^*c*^	5.73 ± 1.77^*b*^	0.47 ± 0.26^*b*^	0.16 ± 0.04^*c*^
Pulp coat	1.32 ± 0.12^*a*^	3.69 ± 1.94^*c*^	0.39 ± 0.04^*b*^	0.14 ± 0.05^*c*^
Ascorbic acid	0.96 ± 0.17^*a*^	2.25 ± 0.89^*d*^	0.55 ± 0.12^*b*^	–
EDTA	–	–	–	0.27 ± 0.09^*b*^

*The radical scavenging abilities of the fruits parts were determined as described and expressed as percentage. The IC_50_ (effective concentration causing 50% antioxidant ability) were calculated using nonlinear regression analysis. Values represent mean ± standard deviation (n = 3). Values with the same letter within a column are not significantly different (*p* > 0.05). Positive control: ascorbic acid and EDTA (Ethylenediaminetetraacetic acid) were used for DPPH*, OH*, NO* and Fe^2+^ chelating ability respectively

### Inhibition of FeSO_4_ and sodium nitroprusside induced lipid peroxidation in the pancreas

**Fig. 2:**
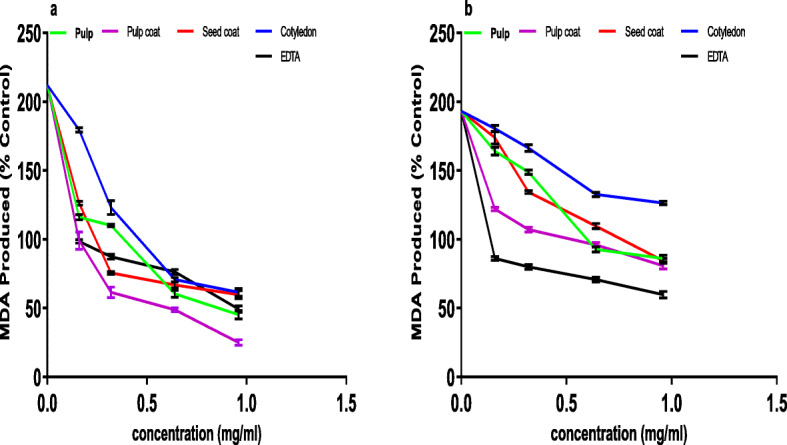
(**a**) Fe^2+^-induced MDA inhibition and (**b**) SNP-induced MDA inhibition of extract from African star apple fruit parts in rat pancreas tissue homogenate. Values represent means ± standard deviation of triplicate readings

The crude extracts from various parts of African star apple fruit led to a dose-dependent significant reduction (*P* < 0.05) in the content of MDA of FeSO_4_-stressed pancreas homogenates (Fig. 2a) with pulp coat (IC_50_ = 0.34 mg/ml) having the greatest inhibitory effect while the seed coat (IC_50_ = 0.68 mg/ml) had the lowest when IC_50_ values were taken into account in Table 4. Similarly, the extract of the fruit parts repressed the production of MDA in the sodium nitroprusside induced pancreatic tissue in a dose-dependent way (Fig. 2b). Also, judging by the IC_50_ value in Table 4, the crude extract of pulp coat had the highest inhibitory effect on sodium nitroprusside-induced lipid peroxidation in rat pancreas when compared to other fruit parts.

**Table 4:** Effective concentration causing 50% antioxidant ability (IC_50_ values) of aqueous extract of different parts of ripe African star apple fruit on inhibition of FeSO4 and SNP-induced lipid peroxidation in rat’s pancreas

Sample	FeSO_4_-Induced lipid peroxidation (mg/ml)	SNP-induced lipid peroxidation (mg/ml)
Pulp	0.54 ± 0.08^*a*^	0.63 ± 0.14^*a*^
Cotyledon	0.64 ± 0.17^*b*^	0.89 ± 0.13^*b*^
Seed coat	0.68 ± 0.15^*b*^	0.91 ± 0.15^*b*^
Pulp coat	0.34 ± 0.07^*c*^	0.49 ± 0.11^*c*^
EDTA	0.48 ± 0.04^*a*^	0.32 ± 0.10^*d*^

The IC_50_ (effective concentration causing 50% antioxidant ability) were calculated using nonlinear regression analysis. Values represent mean ± standard deviation (n = 3). Values with the same letter within a column are not significantly different (p > 0.05). Positive control: EDTA (Ethylenediaminetetraacetic acid)

### Inhibition of *α*-amylase and *α*-glucosidase activities in rat pancreas

The actions of carbohydrate hydrolyzing enzymes (α-amylase and α-glucosidase) were inhibited by the fruits parts in a dose-dependent pattern (Table 5) with pulp coat exhibited the highest inhibition for α-amylase (lower IC_50_ value of 1.39 mg/mL) compared to seed coat (4.16 mg/mL), which had the least (Table 5). Nevertheless, acarbose still had the highest inhibitory effect on α-amylase (Table 5). Similarly, the fruits parts inhibited α-glucosidase activity in vitro in a dose-dependent pattern with the pulp coat had the highest inhibitory effect on α-glucosidase (lower IC_50_ value of 1.35 mg/mL) while cotyledon had the least (2.29 mg/mL) (Table 5). However, the inhibitory effect of the pulp coat was higher when compared with acarbose (1.55 mg/mL) (Table 5).

**Table 5:** Effective concentration causing 50% inhibitory ability (IC_50_ values) of α-amylase and α-glucosidase inhibitory activities of fruits parts

Sample	α-amylase inhibitory activity (mg/ml)	α-glucosidase inhibitory activity (mg/ml)
Pulp	1.46 ± 0.16^*a*^	1.48 ± 0.17^*a*^
Cotyledon	3.96 ± 0.59^*b*^	2.29 ± 0.35^*b*^
Seed coat	4.16 ± 0.62^*c*^	1.79 ± 0.25^*c*^
Pulp coat	1.39 ± 0.14^*d*^	1.35 ± 0.13^*d*^
Acarbose	1.20 ± 0.08^*e*^	1.55 ± 0.19^*c*^

The IC_50_ (effective concentration causing 50% inhibitory ability) were calculated using nonlinear regression analysis. Values represent mean ± standard deviation (n = 3). Values with the same letter within a column are not significantly different (p > 0.05)

### Polyphenolic constituents of African star apple fruit parts

**Fig. 3:**
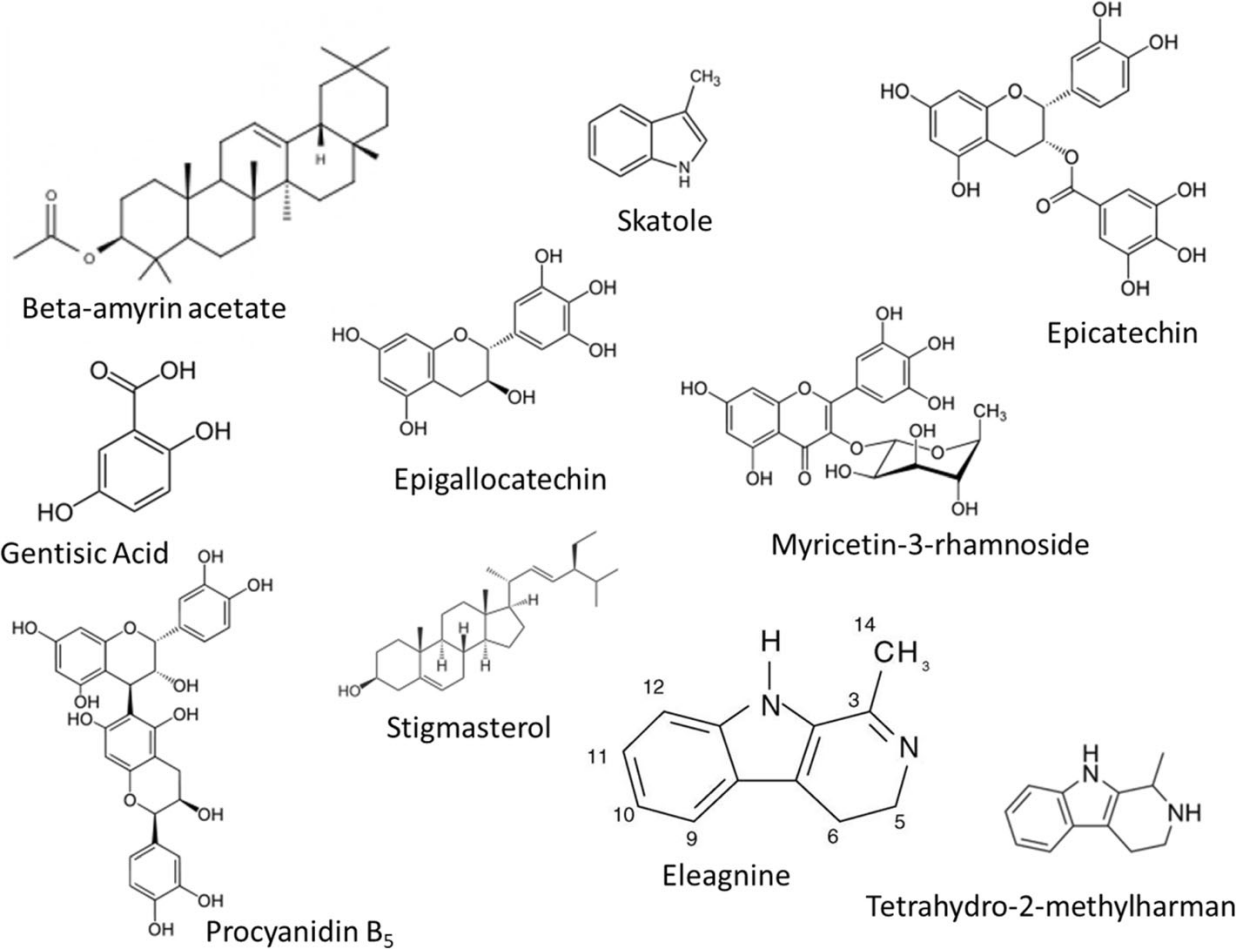
Structures of compounds identified in *Chrysophyllum albidum* fruit parts, Source: ChemSpider

Table 6 presents the HPLC-DAD characterization of polyphenolic constituents contained in the fruit parts. As shown in Table 6, beta-amyrin acetate, eleagine, epicatechin, epigallocatechin, skatole, stigmasterol, and tetrahydro-2-methyl harman are contained in all the fruits while gentisic acid, myricetin-3-rhamnoside, and procyanidin-B_5_ are other phenolic constituents in most of the fruits parts.  The stuctures of the polyphenolic constituents found in the fruit parts are presented in Fig. 3.

**Table 6:** Phenolic components of aqueous extract of African star apple fruit parts

Compounds	African star apple fruit parts			
	Pulp mg/g	Cotyledon mg/g	Seed coat mg/g	Pulp coat mg/g
Beta-amyrin acetate	2.42 ± 0.03a	4.24 ± 0.01a	2.83 ± 0.02a	2.66 ± 0.01a
Eleagnine	0.86 ± 0.01b	0.34 ± 0.03b	1.46 ± 0.01a	1.31 ± 0.01a
Epicatechin	0.98 ± 0.02b	1.39 ± 0.02c	1.27 ± 0.02b	1.24 ± 0.03a
Epigallocatechin	0.78 ± 0.01b	0.34 ± 0.01b	0.97 ± 0.02c	0.87 ± 0.02b
Gentisic Acid	–	0.67 ± 0.02b	0.44 ± 0.02c	0.21 ± 0.01c
Myricetin rhamnoside	0.50 ± 0.02c	–	0.78 ± 0.02c	0.64 ± 0.02b
Procyanidin B_5_	–	–	0.54 ± 0.01c	0.54 ± 0.01b
Skatole	0.84 ± 0.01b	0.32 ± 0.01b	1.59 ± 0.02b	1.49 ± 0.02a
Stigmasterol	3.74 ± 0.02a	2.47 ± 0.02a	5.82 ± 0.02d	5.25 ± 0.02d
Tetrahydro-2-methylharman	1.05 ± 0.01a	0.35 ± 0.01b	1.66 ± 0.03a	1.59 ± 0.03a

Results are expressed as mean ± standard deviations (SD) of three determinations. Averages followed by different letters differ by Tukey test at p < 0.05

## Discussion

The poor awareness and research supporting the intake of fruits and vegetables amid diabetics has experienced low adherence in the developing countries [36]. Consistent intake of fruits has been linked to reduced risks of stroke, cardiovascular disease, cancer, cataracts, neurodegenerative disease, and some of the functional degenerations linked with aging [37]. Phytochemicals, water, fiber, sugars, and vitamin C are usually high in fruits. The process of ripening in fruits resulting from starch hydrolysis to sugar can increase the sugar content in most fruits [38]. Therefore, this could be a possible reason for the low starch and high sugar contents of the fruits parts used for the analysis. This corroborates the findings of Oboh et al. [39] who explained that starch is converted to reduced sugar following fruit ripening. It has been found that in comparison with other similar products which are high in amylopectin, products that are high in amylose induce low insulin responses and blood glucose. In this study, despite the higher content of amylopectin compared to amylose, the fruits parts showed a potentially good response to hyperglycemia. This could be ascribed to other constituents, including fiber and phenolic, which, according to previous studies, could help to lower blood glucose [40, 41]. It has also been reported that compared to foods with low amylose content, food with higher amylose content has lower digestibility [42, 43], and this could be an important justification for the use in aiding digestion.

The estimated glycemic index (eGI) portrays the carbohydrates consumed in diverse food types on the basis of postprandial blood glucose level [43, 44]. Dietary changes are often essential for controlling type-2 diabetes, regardless of the insulin requirement. The eGI was expressed in an effort to help those that are diabetic with their choices of food based on the recommendation that they select foods with a low GI [45]. In this study, the pulp coats which are usually disposed recorded the lowest eGI. Thus, it may be suggested for consumption for those who are diabetic. This result also corroborates the findings of Oboh et al. [39] who reported a low eGI in African star apple fruit. The lowest eGI in the pulp coat could be due to the presence of polyphenols, sucrose, and fibers. Previous studies have also revealed that low-GI diets have the tendency of increasing glycemic control in people who have low tolerance to glucose and type-II diabetes by reducing fasting blood glucose and glycated proteins and also cause insulin sensitivity to improve.

In the onset, Chronic hyperglycemia was implicated as well as the pathogenesis of diabetes and its associated complications [46]. There are signs that chronic hyperglycemia can induce the creation of species with reactive oxygen and eventually result in pancreatic cell damage and oxidative stress [9, 46]. The radical scavenging activities exhibited by the fruit parts observed in this study corroborates the results of Oboh et al. [39] who found that fruits possess antioxidant properties. The radical scavenging activity of the fruit parts has strong relationship with the vitamin C and phenolic contents. Furthermore, past studies have presented ascorbic acid as a strong antioxidant and scavenger of oxygen-determined radicals, for example, hydroxyl (OH) radical and singlet oxygen [47], in spite of the fact that the antioxidant properties of numerous plants with their radical scavenging capacity have been connected to their phenolic content [7, 48]. The finding that sodium nitroprusside (SNP) and iron led to a significant rise in the content of MDA in the pancreas corroborates with earlier findings where iron was found to be a potential initiator of lipid peroxidation [21]. The increase in lipid peroxidation in the presence of iron could be ascribed to the iron’s ability to catalyze one-electron transfer reactions which produce ROS, for instance, the reactive hydroxyl radical that is produced from H_2_O_2_ through the Fenton reaction. Iron also decomposes lipid peroxides, thereby producing alkoxyl and peroxyl radicals, which supports lipid oxidation propagation [49]. In the pancreas, iron accumulates in acinar cells and in the Islets of Langerhans. This leads to the damage of the β-cells related to diabetes mellitus [50]. Thus, the reduction of iron could prompt a reduction in oxidative stress in the entire body [51]. Nevertheless, the crude extracts from the various parts of African star apple fruit led to a dose-dependent reduction (which was significant at 5%) in the content of MDA of the SNP and Fe^2+^-stressed pancreas homogenates. It is difficult to categorically state the means by which Fe^2+^-induced lipid peroxidation is inhibited. However, it is possible for the water extractable phytochemicals to have formed complexes with the iron, thus stopping them from catalyzing the instigation of lipid peroxidation and/or it is also possible for the phytochemical to have scavenged the free radical generated by the Fe^2+^-catalyzed reaction [21]. Glucose, in the presence of transition metals (e.g. Fe), can be oxidized to produce ROS, which has been implicated in the advancement of diabetic complications as well as diabetes itself. Excess cytosolic iron is a common factor of gestational diabetes [52] and a vivid risk factor for the disease in normal populations [53]. Insulin sensitivity is improved by lowering iron [53]. Therefore, the status of iron may be a major factor in the development of type II diabetes [53]. There is also a possibility for Iron to be included in the Fenton reaction which leads to the production of hydroxyl radical. These radicals subsequently attack DNA, protein, membrane lipids and many other biomolecules which are important physiologically.

Hyperglycemia is caused by degradation of foods which are starchy by carbohydrate-hydrolyzing enzymes (such as α-glucosidase and α-amylase). The common therapeutic approaches applied in treating and managing diabetes includes using synthetic inhibitors of α-glucosidase and α- amylase activities to slow the rate at which glucose is released into the blood stream [39]. According to Saito et al. [54], using these synthetic inhibitors (such as voglibose and acarbose) presents some side effects like flatulence, meteorism, and abdominal cramps. Managing diabetes taking a dietary approach by using plants that have natural inhibitors of α- glucosidase and α-amylase is more advantageous. Our findings indicate that the fruit parts also displayed their ability to inhibit α- glucosidase and α-amylase activities in vitro. However, of all the fruit parts, the pulp coat indicated the highest inhibitory activity on α-amylase. Likewise, a similar trend was found for α-glucosidase inhibitory activity. Studies have shown fruits to have various health benefits, with antidiabetic effect inclusive [55, 56].

The antioxidant qualities of plant foods have been associated with the presence of an array of important phenolic and non-phenolic phytochemicals such as alkaloids, phenolic acids, and flavonoids [57, 58]. However, classification of the extracts with HPLC showed that the major constituent of the pulp, cotyledon, seed coat and pulp coat of ripe African star apple fruit are Beta-amyrin acetate, eleagine, epicatechin, epigallocatechin, skatole, stigmasterol and tetrahydro − 2- methyl harman (Table 6). Historically, phenolic fractions of plants are recognized as inhibitors of carbohydrate hydrolyzing enzymes in higher animals. Phenolic compounds derived from raspberries, red cabbage, and strawberries are recognized as inhibitors of α-glucosidase and α-amylase [55]. Though Jenkins et al. [44] confirmed that as far as managing diabetes is concerned, the notion of glycemic index is not new and adoption of pharmacologic methods to slow the absorption of carbohydrate, most especially using glycoside inhibitors, are currently recognized in diabetes management, it is worth noting that the current study was able to elucidate the eGIs, antioxidant properties as well as α-amylase and α-glucosidase inhibitory properties of Africa star apple fruit parts. In this regard, the study established a connection between the eGI and the hypoglycemic potential of fruit parts, affirming that the fruit parts will be excellent inhibitors of starch hydrolyzing enzymes due to their low eGI and hence possess great potentials in the management of Diabetes Mellitus.

## Conclusion

African star apple fruit parts (pulp, cotyledon, seed coat and pulp coat) exhibited significant antioxidant activities as revealed by their ability to scavenge free radicals as well as their effective ferric reducing power. The low glycemic indices, and the inhibitory effects of the fruit parts on carbohydrate hydrolyzing enzymes (α-amylase and α-glucosidase) due to their phytochemical constituents showed that the fruit parts can be of great benefit as functional dietary supplement to manage or control diabetes-related disorders. Overall, African star apple pulp coat exhibited superior properties and may serve as an important food supplement for health promotion. Hence, further studies on the toxicity studies on different parts of the fruit used in this study should be carried out to ascertain their safety.

## Data Availability

The datasets used to analyze during this study are available from the corresponding author on reasonable request.

## Abbreviations

ABTS: 2, 2′-azino-bis (3-ethylbenzothiazoline-6-sulphonic acid)
DM: Diabetes Mellitus
eGI: Estimated glycemic index
Fe^2+^: Iron (II) chelating ability
NO: Nitric oxide
OH: Hydroxyl
SNP: Sodium nitroprusside
